# Nausea

**Published:** 1884-04

**Authors:** 


### NAUSEA.

Dr. F. Douglas, of Fenton, Mich., says: "For many years I have known that a few drops of cold water thrown in the face would instantly remove nausea, at least for a short time. Many times I have used the idea in taking impressions of the mouth. A few days ago, while administering ether, I saw a nice prospect of a vomiting spell. After the patient began retching severely, I threw a little cold water in the face. The retching instantly ceased, and by repeating it a few times vomiting was prevented, until I had extracted fifteen roots. A day or two afterwards I had a similar case with the same results. The two patients were apparently beyond consciousness."

There is a physiological reason for this. The fifth pair of nerves, so largely distributed to the face, is one of great excito-motor capacity. It is well known that in cases of syncope, the dashing of water in the face excites the respiratory tract. Garretson states that it presides remarkably over both salivary and oro-nasal mucous secretions. By its primary action upon the pharyngeal muscles, and its secondary influence upon the digestive tract, it may well be believed that its excitation, through its terminal filaments, can exercise a control, more or less complete, to prevent nausea.

